# Investigation of SHOX Gene Mutations in Turkish Patients with Idiopathic Short Stature

**DOI:** 10.4274/jcrpe.2307

**Published:** 2016-06-06

**Authors:** Kenan Delil, Halil Gürhan Karabulut, Bülent Hacıhamdioğlu, Zeynep Şıklar, Merih Berberoğlu, Gönül Öçal, Ajlan Tükün, Hatice Ilgın Ruhi

**Affiliations:** 1 Marmara University Faculty of Medicine, Department of Medical Genetics, İstanbul, Turkey; 2 Ankara University Faculty of Medicine, Department of Medical Genetics, Ankara, Turkey; 3 Süleymaniye Maternity Training and Research Hospital, Clinic of Pediatric Endocrinology, İstanbul, Turkey; 4 Ankara University Faculty of Medicine, Department of Pediatric Endocrinology, Ankara, Turkey

**Keywords:** Idiopathic short stature, SHOX gene, pseudoautosomal region 1, height, arm span-height difference

## Abstract

**Objective:**

The frequency of mutations in the short stature homeobox (SHOX) gene in patients with idiopathic short stature (ISS) ranges widely, depending mostly on the mutation detection technique and inclusion criteria. We present phenotypic and genotypic data on 38 Turkish patients with ISS and the distinctive features of 1 patient with a SHOX deletion.

**Methods:**

Microsatellite markers (MSMs) DXYS10092 (GA repeats) and DXYS10093 (CT repeats) were used to select patients for fluorescent in situ hybridisation (FISH) analysis and to screen for deletions in the SHOX gene. The FISH analysis was applied to patients homozygous for at least one MSM. A Sanger sequencing analysis was performed on patients with no deletions according to FISH to investigate point mutations in the SHOX gene.

**Results:**

One patient (2.6%) had a SHOX mutation.

**Conclusion:**

Although the number of cases was limited and the mutation analysis techniques we used cannot detect all mutations, our findings emphasize the importance of the difference in arm span and height when selecting patients for SHOX gene testing.

**WHAT IS ALREADY KNOWN ON THIS TOPIC?**

The frequency of mutations in the short stature homeobox (SHOX) gene in patients with idiopathic short stature ranges widely, depending mostly on the mutation detection technique and inclusion criteria.

**WHAT THIS STUDY ADDS?**

Short children should be carefully investigated with respect to these mutations, even if they have only mildly disproportionate stature.

## INTRODUCTION

Idiopathic short stature (ISS) is defined as a condition where a person’s height is more than two standard deviations (SDs) below the average height for a specific age, gender, and population with no other systemic, endocrine, nutritional, or chromosomal abnormalities, nor a history of intrauterine growth retardation and low weight for gestational age (1,2). ISS excludes other identifiable conditions not based on positive specific signs of ISS.

Height has a high degree of heritability and is a polygenic quantitative trait that shows complex and monogenic Mendelian inheritance patterns (3). One study reported that hundreds of variants clustered in specific genomic loci play roles in the human height trait (4). A clearly relevant gene that strongly affects height is the short stature homeobox (SHOX) gene, mapped to pseudoautosomal region 1 (PAR1) of the X and Y chromosomes. The SHOX gene has been reported to cause ISS and the short stature seen in patients with Turner’s syndrome, Leri-Weill dyschondrosteosis, and Langer mesomelic dysplasia (5,6,7,8). A high recombination rate in PAR1 is associated with mandatory crossover between the X and Y chromosomes during meiosis (9,10,11). All 24 genes in the PAR1 region escape X inactivation (12). As a result, all genes located in the PAR1 region have two functional copies in humans and show a pseudoautosomal inheritance pattern (10,13). The only gene in the PAR1 region clearly associated with a disease is SHOX (14).

The frequency of mutations in the SHOX gene in patients with ISS varies widely, depending mainly on the mutation detection technique and inclusion criteria. In one study, approximately 2.4% of a large cohort of patients with ISS had SHOX mutations, of which 80% were complete gene deletions (15). Stuppia et al (16) reported a 12.5% frequency of SHOX mutations in 56 patients with ISS.

In this study, we evaluated the frequency of mutations in the SHOX gene in patients with ISS and discussed the distinctive clinical and radiological features of patients with such mutations.

## METHODS

The study was approved by the Ethics Committee of the Ankara University Faculty of Medicine. Written informed consent was obtained from all patients and their legal guardians. In all, 38 patients (34 females and 4 males; mean age, 11.84 years; range, 6.5-17 years) were included in the study. We used the following criteria based on the definition of ISS: height <-2 SD of the mean height for a given age, sex, and population group; normal karyotype (for girls); no evidence of chronic disease (e.g., chronic renal failure, chronic anaemia, celiac disease, malabsorption, malnutrition, chronic hepatic disease, chronic infectious disease, or congestive heart failure); no growth hormone (GH) deficiency and/or GH resistance based on the routine provocation test (peak GH>10 ng/mL) and normal insulin-like growth factor-1 level; no history of low birth weight; and no apparent skeletal disease.

The clinical assessment included measurements of height, weight, and sitting height, as well as the lengths of the upper segment (US), lower segment (LS), forearm, upper arm, hands, and feet. Furthermore, the degree of short stature, US/LS ratio, difference between arm span and height, assessed body proportions, extremities/trunk ratio (ETR; sum of leg length and arm span divided by sitting height), relative body mass index (RBMI), and the presence of additional features (e.g., appearance of muscular hypertrophy, cubitus valgus, forearm bowing) were evaluated.

### Mutation Analysis

**Figure 1:**
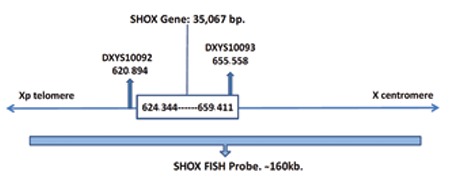
Schematic presentation of SHOX gene, SHOX fluorescent in situ hybridisation probe, and microsatellite markers DXYS10092 and DXYS10093 (according to Human GenomeAssembly GRCh38). FISH: fluorescent in situ hybridisation

Genomic DNA was extracted from 1 mL peripheral blood using the Magna Pure LC instrument (Roche Applied Science, Manheim, Germany). We used an approach similar to the study of Chen et al (17) in which microsatellite markers (MSMs) were used to select patients for multiplex ligation-dependent probe amplification (MLPA) analysis to screen deletions in the SHOX gene. We used DXYS10092 (GA repeats) and DXYS10093 (CT repeats) to select patients for fluorescent in situ hybridisation (FISH) analysis to screen for SHOX gene deletions (Figure 1). Benito-Sanz et al (18) reported heterozygosity values of 0.96 and 0.69 for DXYS10092 and DXYS10093, respectively, and the repeat ranges were 18 and 14, respectively. Both MSMs were amplified by polymerase chain reaction and analysed on 8% polyacrylamide gels (see Supplementary Material).

The FISH analysis was applied to patients homozygous for at least one MSM using lymphocyte metaphase spreads and the Aquarius SHOX probe (cat no: LPU 025; Cytocell, Cambridge, UK).

Sanger sequencing was applied to patients with no deletions detected by the FISH analysis to investigate point mutations in exons 2, 3, 4, 5, and 6a and their exon-intron junction sites in the SHOX gene (see Supplementary Material).

## RESULTS

**Figure 2:**
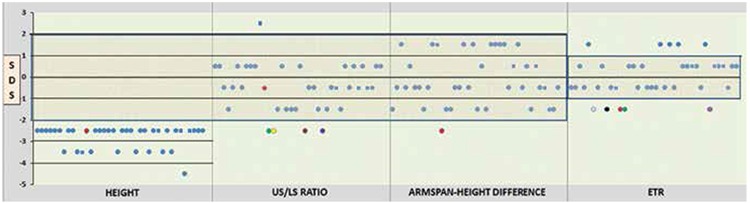
Height, upper segment/lower segment ratio, arm span-height difference and extremities-trunk ratio representations together with standard deviation score for all patients. Males are illustrated by square, whereas females by round. Patients lined up in order to patient number from left to right. Grey colour for P6, black for P9, red for P12, green for P13, yellow for P14, brown for P21,purple for P25, pink for P32. US: upper segment, LS: lower segment, ETR: extremities-trunk ratio

In all, 36 index cases and an additional two children (patient 2 was a monozygotic twin brother of patient 1, and patient 34 was a sister of patient 33) were evaluated. All patient heights were <-2 SD (Figure 2). Mean height SD was -2.76±0.46. Height measurements and additional anthropometric data are shown in Figure 2 and Table 1.

**Figure 3:**
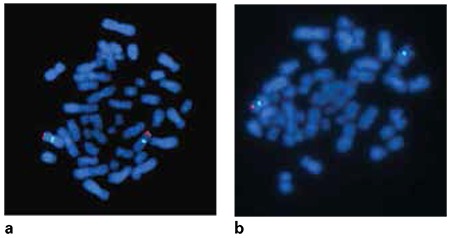
(a and b) Fluorescent in situ hybridisation images from P12 and P20. P20 showed two blue and two red signals meaning normal female. P12 showed two blue but 1 red signal meaning SHOX gene deletion (right). Probe specification: SHOX probe; Xp22.33/Yp11.2, (Red)/DYZ1 probe; Yq12, (Green) and DXZ1 probe; Xp11.1-q11.1, (Blue)

**Figure 4:**
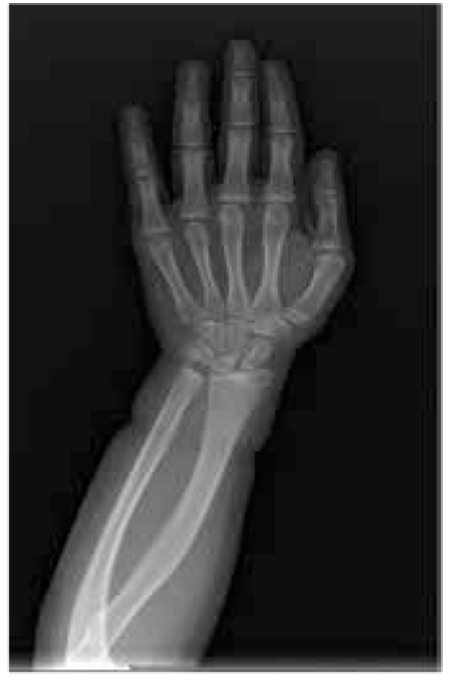
Hand and forearm radiography of the patient with SHOX deletion (P12) showing minimal bowing and mild wedging of the radius

One patient (2.6%, patient 12) had a SHOX deletion detected by FISH analysis (Figure 3). Patient 12 was an 11.5-year-old girl. She had a sister and two brothers with normal height, and her parents were first cousins. Her mother’s height was 153 cm and the father’s height was 178 cm. The mother’s SHOX FISH analysis was normal. Patient 12’s main clinical findings were short stature (height, 137 cm; -2.02 SD), disproportionate body measurements (arm span/height difference: -7, <-2 SD), obesity (RBMI, 126.1%), short forearms, cubitus valgus, muscular hypertrophy, genu valgus, micrognatia, high palate, and bilateral epicanthus. Hand and forearm radiography of the patient showed minimal bowing and mild wedging of the radius (Figure 4).

## DISCUSSION

GH treatment is quite effective for patients with ISS and a mutation in the SHOX gene (19). Thus, it is important to demonstrate genetic aetiology in these cases. The frequency of mutations in the SHOX gene in patients with ISS is 2-15% (15,16,20,21,22,23). According to our results, this frequency was 2.6% in children with ISS.

Rappold et al (15) screened intragenic mutations using single-strand conformation polymorphism analysis in 900 patients followed by sequencing of 750 patients and detected 3 patients (0.4%) with functional mutations. They also analysed complete gene deletions using FISH in 150 patients and detected 3 patients (2%) with deletions. Another study on 56 patients with ISS reported a 12.5% (n=7) frequency of SHOX mutations (16). Jorge et al (21) reported a rate of 3.2% (2/63 patients with ISS). A large study that included 1534 patients with ISS reported a rate of 2.2% (n=34) (22). This wide range is mainly due to the mutation detection technique and the case inclusion criteria. Our results are compatible with the findings in these studies.

The clinical expression of SHOX deficiency is highly variable, as short stature is frequently nonspecific in preschool children. SHOX deficiency is more severe in females than males. Young children with SHOX deficiency may not have any specific clinical findings, but the phenotype usually becomes more pronounced with age, and characteristic signs appear over time (21,24,25). The most prominent features besides short stature are a Madelung deformity, short fourth and fifth metacarpals, high arched palate, increased carrying angle of the elbow, scoliosis, and micrognathia.

Rappold et al (22) investigated the presence of SHOX defects in a large cohort of 1608 children with short stature. The mean SD in height was not different between the participants with short stature with or without identified defects in the SHOX gene in that study. The authors created an evidence-based scoring system based on the clinical features of 68 patients with SHOX defects to identify the most appropriate children for testing. They concluded that some clinical findings were useful as clues to distinguish patients with a SHOX mutation among patients with short stature and that the presence of any combination of reduced arm span/height ratio, increased sitting height/height ratio, above average body mass index (BMI), a Madelung deformity, cubitus valgus, short or bowed forearms, dislocation of the ulna at the elbow, or muscular hypertrophy should prompt the clinician to conduct a molecular analysis for the SHOX gene. An increased sitting height/height ratio, above average BMI, cubitus valgus, short forearms, and muscular hypertrophy were noted in our case with an SHOX gene deletion.

Binder et al (24) used ETR to select patients more likely to have a SHOX mutation. They suggested that screening for SHOX mutations should be limited to patients whose ETR is <1.95 + ½ height (m) and close inspection of a hand radiograph to detect the main characteristics of SHOX deficiency (pyramidalisation of the carpal row, radiolucency of the distal radius at the ulnar border, and triangularisation of the distal radius) in school-age children. Jorge et al (21) confirmed the usefulness of this approach and recommended using the sitting height/height ratio because it is easier to use than ETR. Our results suggest that the ETR and the difference in arm span and height are useful parameters. The US/LS ratio was not reliable alone, as this parameter was normal in our patients (Figure 2).

A radiographic examination of a patient with an SHOX gene mutation may demonstrate abnormal carpal wedging, triangularisation of the distal radial epiphysis, radial lucency, shortening of fourth and fifth metacarpals, and radial bowing (26). We did not detect any striking findings on a radiograph of the left hand in our patient, and she had only minimal bowing of the radius and mild wedging. It is not possible to analyse every child with ISS for a SHOX gene mutation because of its low incidence. Phenotypic variation in short children can affect the decision to perform a genetic analysis. Beyond the typical dysmorphic signs, a positive family history, careful anthropometric measurements and an x-ray evaluation of the hand and wrist can be used to support this decision.

Although we had a limited number of cases and the mutation analysis techniques used could not detect all mutations, our findings emphasize the importance of the difference between arm span and height when selecting patients for SHOX gene testing. Nevertheless, more extensive studies with larger groups of patients and a wider range of mutation screening techniques are needed.

Deletions are the most frequently detected SHOX gene mutations (15). In our study, we first performed MSM and then a FISH analysis to screen for SHOX gene deletions. Funari et al (27) suggested that MLPA should be the first molecular method used to screen for SHOX gene deletions. We also suggest using MLPA first because SHOX deletions are highly heterogeneous, so numerous MSM loci may need to be studied, and MLPA can detect smaller deletions than FISH.

In summary, our patient with a SHOX mutation had no obvious findings associated with such a gene deletion. She had a disproportionate body, which could easily go unnoticed, but she had no obvious Madelung deformity.

In conclusion, we detected an SHOX gene deletion in 1 of 38 children with ISS. Short children should be carefully investigated with respect to these mutations, even if they have only mildly disproportionate stature.

## Ethics

Ethics Committee Approval: The study was approved by the Ethics Committee of the Ankara University Faculty of Medicine Ankara University, 04/10/2010, Informed Consent: It was taken.

Peer-review: External peer-reviewed.

## Figures and Tables

**Supplementary Material:**
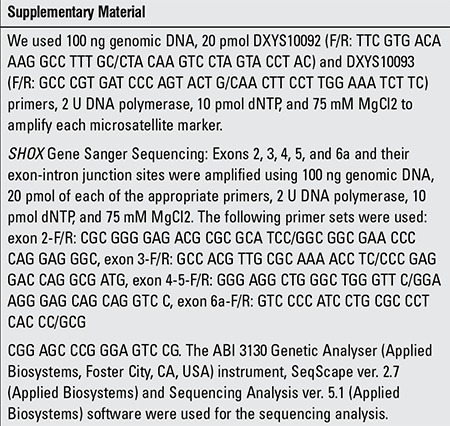
Supplementary Material

**Table 1:**
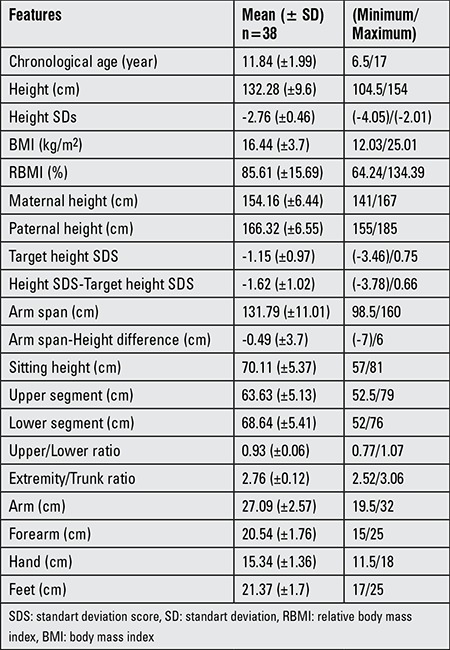
Evaluation of anthropometric measurements in the patients
